# Whole Genome Sequencing: Bridging One-Health Surveillance of Foodborne Diseases

**DOI:** 10.3389/fpubh.2019.00172

**Published:** 2019-06-27

**Authors:** Peter Gerner-Smidt, John Besser, Jeniffer Concepción-Acevedo, Jason P. Folster, Jasmine Huffman, Lavin A. Joseph, Zuzana Kucerova, Megin C. Nichols, Colin A. Schwensohn, Beth Tolar

**Affiliations:** ^1^The Enteric Diseases Laboratory Branch, Centers for Disease Control and Prevention, Atlanta, GA, United States; ^2^The Outbreak Response and Prevention Branch, Centers for Disease Control and Prevention, Atlanta, GA, United States

**Keywords:** whole genome sequencing (WGS), outbreak, one health, zoonotic, food, environment, animals, investigation

## Abstract

Infections caused by pathogens commonly acquired from consumption of food are not always transmitted by that route. They may also be transmitted through contact to animals, other humans or the environment. Additionally, many outbreaks are associated with food contaminated from these non-food sources. For this reason, such presumed foodborne outbreaks are best investigated through a One Health approach working across human, animal and environmental sectors and disciplines. Outbreak strains or clones that have propagated and continue to evolve in non-human sources and environments often show more sequence variation than observed in typical monoclonal point-source outbreaks. This represents a challenge when using whole genome sequencing (WGS), the new gold standard for molecular surveillance of foodborne pathogens, for outbreak detection and investigation. In this review, using recent examples from outbreaks investigated in the United States (US) some aspects of One Health approaches that have been used successfully to solve such outbreaks are presented. These include using different combinations of flexible WGS based case definition, efficient epidemiological follow-up, traceback, surveillance, and testing of potential food and environmental sources and animal hosts.

## Introduction

Infections caused by pathogens commonly transmitted by food are common, potentially all preventable and therefore of major public health importance. They are a problem all over the world affecting all parts of society in developing and developed countries (1). Although mostly presenting as a self-limiting diarrheal illness, more severe illness requiring hospitalization is frequently seen. Foodborne illness caused by certain pathogens, e.g., *Listeria monocytogenes*, carry a significant mortality. Outbreaks are common with ~1,000 outbreaks being investigated in the US every year (2). Foodborne pathogens can be any infectious agent, e.g., bacteria, parasites, virus, and prions, even though this review focuses on bacterial pathogens.

In the US approximately one in six persons acquires a foodborne illness every year (3). However, it needs to be kept in mind that not all infections caused by pathogens commonly transmitted through food are actually foodborne. Although illness is often caused through ingestion of contaminated food, the primary reservoir of these pathogens is rarely food but rather animals, water or the environment. The reservoir of pathogens like non-typhoidal *Salmonella, E. coli, Campylobacter*, and *Yersinia* is primarily zoonotic, i.e., wildlife, pets, or food production animals. *Listeria monocytogenes* is ubiquitous and may be found in the environment, animals and food. A classical example of a waterborne pathogen is *Vibrio* spp. but many foodborne enteric pathogens may also be transmitted through contaminated recreational or drinking water. Ill humans can also infect each other. Thus, infection caused by pathogens commonly transmitted by food is a classic example of a One-Health challenge. The One Health concept includes the health of humans, animals and the environment. In this paper, the focus is on human infections. If public health investigators only focus their attention to food sources and vehicles when investigating potential foodborne outbreaks, they will miss opportunities to identify primary sources and prevent further illness and outbreaks from animal or environmental sources. Even when the vehicle is foodborne, e.g., meat from a specific supplier, a proper conducted investigation should include a root cause analysis. For example, in addition to removing a vehicle from the market, a thorough trace-back of the vehicle to the primary production should be performed, e.g., to the farm and the suppliers of that farm, even if the ultimate source is in a different country or on a different continent. This can best be achieved through a One Health approach to investigation working across human, animal, and environmental sectors and disciplines.

With the introduction of affordable and fast next generation sequencers in the early 2000s, WGS has revolutionized molecular epidemiology and laboratory surveillance of infections caused by pathogens commonly transmitted though food providing public health researchers with a tool of unprecedented precision and discrimination for subtyping. Additionally, WGS may provide a wealth of information at the push of a button that exceeds what in the past was typically gathered using traditional phenotypic and genotypic tests in public health laboratories e.g., species identification, serotype, pathotype, virulence profile, antimicrobial resistance, and plasmid content to name a few. A description of the analytical tools is beyond the scope of this paper and may be found elsewhere (4–6). Using WGS, public health scientists typically detect outbreaks by looking for tight clusters of infections caused by a specific pathogens in time and space typically differing by <10 single nucleotide polymorphisms (SNPs) or 10 alleles by core genome multi-locus sequence typing (cgMLST) analysis (7). This is the typical scenario of a monoclonal outbreak from a point source that has been contaminated because of a single event (8–12). However, in many outbreaks with a zoonotic or environmental source, the outbreak strains have persisted in their hosts and reservoirs and therefore have time to diversify beyond what is expected in a point source outbreak (13, 14). In such outbreaks, the source may also be contaminated with more strains leading to polyclonal, possibly multi-species outbreaks. Detecting and investigating such outbreaks pose specific challenges. In this paper, a number of such outbreaks that recently have been investigated in the US will be reviewed with an emphasis on their characteristics as experienced with WGS using the cgMLST subtyping approach used by PulseNet, the US molecular subtyping network for foodborne disease surveillance (15), and how the challenges of their interpretation was overcome.

## A Persistent Polyclonal Outbreak of Listeriosis Associated With Contamination of Ice Cream Production Premises

**Figure 1:**
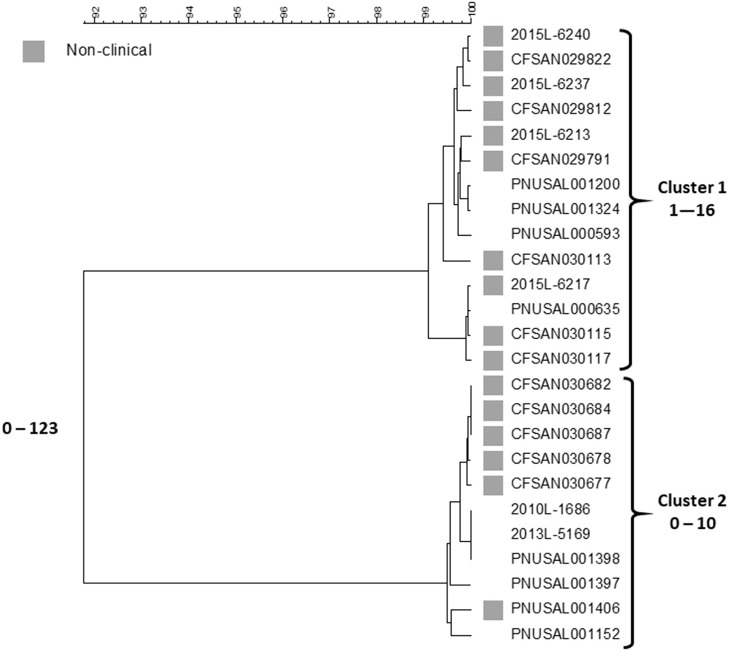
cgMLST UPGMA tree of Lineage I isolate sequences the *Listeria* outbreak linked to ice cream. All clinical isolates and a representative sample of non-clinical product and production environment isolates are included in the tree. The range of allele differences are indicated at the branches of the tree and for clusters to the right of the tree.

In 2015, *Listeria monocytogenes* was isolated from a number of samples of ice cream from a distribution center (https://www.cdc.gov/listeria/outbreaks/ice-cream-03-15/index.html). Some of these isolates matched four clinical isolates from a single hospital in Kansas collected during the past year by PFGE and WGS; a fifth clinical case in the hospital was infected with an unrelated strain. The particular brand of ice cream was regularly served in milkshakes at the hospital and all cases were considered nosocomially acquired. This led to the inspection of the company's production facilities in three states by local authorities and the Food and Drug Administration (FDA) over the next months. Numerous samples from the production facilities and products from two states were positive for *Listeria* in low numbers (16). All of the new isolates were compared against the PulseNet database using PFGE and WGS. Five clinical isolates from patients in three states matched product or production environment isolates by WGS spanning the years 2010–2014. Researchers from FDA compared the sequences of 137 food and environmental and nine clinical isolates (17). This analysis included the four clinical isolates from the hospital outbreak that matched any food or environmental isolates. The isolates represented 13 PFGE patterns but were clustered in only two groups by SNP analysis, one corresponding to the hospital cluster in Kansas and the other containing the historical clinical isolates from three states. All isolates belonged to sequence type (ST) 5 of clonal complex 5 (CC5) of lineage I, molecular serogroup IIb (serotypes 1/2b, 3b, or 7). The isolates within the clusters differed from each other by up to 29 SNPs and between each cluster by 40–52 SNPs. A summary of the Centers for Disease Control and Prevention's (CDC) cgMLST analysis with representative isolate sequences of the outbreak isolates using the PulseNet customized version of the Pasteur scheme (18) is shown in Figure 1.

The fifth Kansas hospital isolate is not included in the figure since it belongs to a different lineage, ST and serotype, lineage II, ST573 and serotype 1/2a, and differs by 1,290–1,377 alleles from any other outbreak isolate. This analysis are generally consistent with the FDA SNP analysis (17). Two clusters are seen, one containing the four Kansas hospital patient isolates and food and environment isolates from one plant and the other the five historical clinical isolates and the non-human isolates from the other facility. Isolates in each cluster differ from each other by up to 16 and 10 alleles, respectively. The two clusters differed by up to 123 alleles. The genetic differences observed within each cluster is slightly higher than typically is observed for point source outbreaks. However, the allele differences between the two clusters were twice as high than observed in the FDA SNP analysis and an average number of allele differences of more than 100 alleles are higher than typically observed between isolates that could be related epidemiologically (7). This speaks against the hypothesis of a recent common origin of the two clones. By phenotypic serotyping the isolates in the Kansas hospital cluster was 1/2b whereas those in the “historical cluster” was 3b. Sequence types (from 7 house-keeping gene MLST) (19) associated with serotype 1/2b strains often also contain serotype 3b strains (CDC, unpublished observation) so although we have never observed two *Listeria* serotypes in a tight monoclonal outbreak it is possible that strains of serotype 1/2b may evolve to serotype 3b or *vice versa*. Although no attempt has been made to use the data as a “molecular clock” to characterize the divergence of the two clusters in this clone, it is not impossible that the clusters could have originated from the same strain at some point in the fairly recent past. It could have been introduced in the two plants at the same time or first in one facility and then shortly thereafter from the first facility to the second. The strains may then have diversified further in each plant. Even though the products seem to have been almost uniformly contaminated (16), the contamination levels in the products were so low to rarely cause disease. Such “low and slow” outbreaks, i.e., outbreaks that go on for a long time with clinical cases occurring within long intervals, could not be detected or were not further pursued in the past because of the poorer resolution of PFGE. With the superior resolving power of WGS, this has now changed. This challenges the time aspect of a typical outbreak investigation, i.e., a cluster of clinical illness in space and time. A typical monoclonal point source outbreak evolves quickly over days to a few months. However, this outbreak shows that the time aspect of the clustering may be much longer, i.e., years. This outbreak is also noteworthy for two other aspects: (1) both clusters were detected by matching food/environmental isolates to clinical cases, and (2) the diversity by PFGE was higher than observed by WGS; at least 16 different PFGE profiles were observed by PulseNet, whereas WGS indicated that two possibly related clones caused it with one case patient harboring a third unrelated strain. It is well-known that PFGE diversity is driven by loss or acquisition of mobile genetic elements and not by mutations. In their study of this outbreak, Chen et al. (17) observed that loss or gain of prophages could explain some of the PFGE variations. Such gains and losses typically occur during long term *in vivo* propagation of a strain and therefore supports the notion that the outbreak strain evolved over the years and it likely was present in the production plants. Gains and losses of mobile genetic elements are usually not reflected in a SNP or cgMLST analysis since such sequences are often filtered out before analysis because they distort the phylogenetic signal.

## A Persistent Polyclonal Multi Drug Resistant Outbreak of *Salmonella* ser. Heidelberg Linked to Chicken From Single Production Company

This outbreak was investigated using PFGE, the PulseNet primary subtyping method at the time it happened. After the outbreak was over, WGS was conducted on a small sample of 30 isolates representing all PFGE patterns and sources, and representative antimicrobial susceptibilities (https://www.cdc.gov/salmonella/heidelberg-10-13/index.html). The investigation began after a cluster of infections caused by *Salmonella* ser. Heidelberg of a rare PFGE pattern (PulseNet pattern JF6X01.0258) was detected by PulseNet in 2013 (20). At the same time, a chicken breast retail sample from a production company A cultured positive for the same strain. During a few months following the detection of the outbreak, six additional clusters of clinical isolates were identified. Some of the PFGE patterns in these clusters were similar to the original outbreak pattern (differing by up to three bands) and since the patients clustered in time, geographic distribution and food history with patients from the first cluster, all seven clusters were merged into the investigation. Six out of seven outbreak strains were found in left-over raw chicken from patient homes and from products from three production establishments of company A. A total of 634 outbreak related patients were identified in 29 states and Puerto Rico. Antimicrobial susceptibility testing of patient and product isolates showed numerous profiles with isolates being pan-susceptible, resistant to one, two, three, or more classes of antimicrobials with weak correlation between resistance profile and PFGE pattern. Following recalls and operational adjustments at company A, the outbreak was declared over a year later.

**Figure 2:**
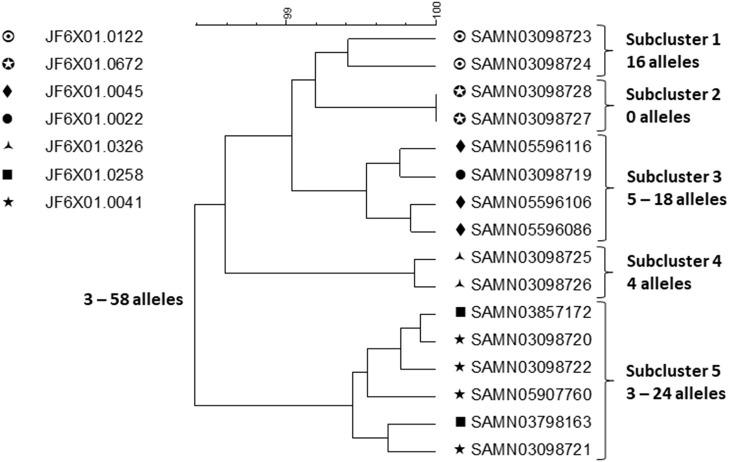
cgMLST UPGMA tree of a representative sample of sequences of *Salmonella* ser. Heidelberg isolates displaying the full WGS diversity and representing the seven PFGE patterns from the outbreak associated with chicken produced by Company A. The range of allele differences are indicated at the branches of the tree and subclusters to the right of the tree.

A small sample of outbreak related isolates from patients with exposure to chicken from company A and product samples were sequenced to shed further light on the outbreak strains (Figure 2).

Clustering was performed by cgMLST using the PulseNet scheme, which contains the same loci as the Enterobase scheme (21). Isolates of the same PFGE pattern clustered together but two subclusters (subclusters 3 and 5) contained isolates with two PFGE patterns intermingled. Food isolates intermingled with patient isolates of the same PFGE pattern by WGS. Considering the whole outbreak cluster, isolates differed by up to 58 alleles and within each subcluster by up to 24 alleles. Using ResFinder (22) and PlasmidFinder (23), 14 different resistance determinants, conferring resistance to seven different drug classes, were identified; eight different plasmid types were identified including common multi drug resistance plasmids, e.g., IncHl2, Incl1, and IncA/C2 confirming the diversity observed by the other method. Due to the small sample of isolates that were sequenced, the sequence variation was likely underestimated. *Salmonella* ser. Heidelberg is commonly associated with chicken. In this outbreak, the outbreak strains had probably been present in the production system for long time, likely years, giving them ample time to diversify and acquire/lose plasmids and with them resistance determinants. It is likely that fewer case-patients would have been recognized in this outbreak if cgMLST alone had been used to detect and delineate it because of the high sequence diversity among subclusters displaying the same PFGE pattern. Thus, this is an example of an outbreak where a subtyping method with poorer discrimination than WGS, i.e., PFGE, better identifies its full extent. It is likely that by WGS small subclusters of highly similar isolates e.g., associated with restaurant or other local events, would have been identified and perhaps linked to products from company A. However, linking them all together and identifying other outbreak related ser. Heidelberg isolates among the large background of sporadic infections caused by this serotype, would be a daunting if not an impossible task.

## A *Salmonella* Outbreak Involving Six Serotypes Associated With Consumption of a Herbal Supplement From South East Asia

**Figure 3:**
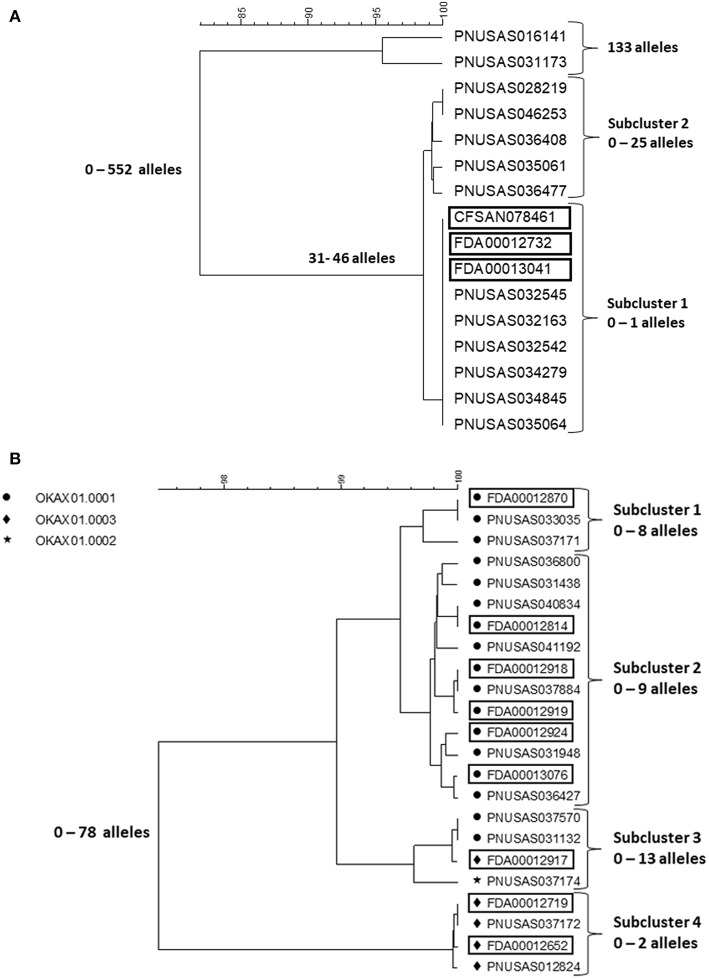
cgMLST UPGMA trees of representative samples of sequences of *Salmonella* ser. I 4,[5],12:b:- **(A)** and Okatie **(B)**. Isolates of ser. I 4,[5],12:b:- all displayed the same PFGE pattern. Isolates of ser. Okatie displayed three PFGE as indicated on the figure. Product isolate identifiers are indicated with rectangles around them. The range of allele differences are indicated at the branches of the tree and subclusters to the right of the tree.

In early 2018, a tight cluster of *Salmonella* ser. I 4,[5],12:b:- (monophasic Paratyphi B var. L(+) tartrate+ [formerly Java]) was identified by PulseNet (https://www.cdc.gov/salmonella/kratom-02-18/index.html). The investigation soon confirmed it as an outbreak and pointed to an unusual vehicle, an opiod agonistic herbal supplement, kratom (*Mitragyna speciose* also known as thang, kakuam, thom, ketum, and biak) sold as powder, capsules, or tea. Leftover and unopened kratom products were tested by local authorities and FDA for *Salmonella* contamination and a number of different serotypes were identified. The outbreak strain was confirmed in the product by PFGE and WGS. A search of the PulseNet national database identified potential case patients infected with some of these additional serotypes, including *Salmonella* ser. Heidelberg, Javiana, Okatie, Thompson, and Weltevreden dating back to the beginning of 2017. Among these serotypes, Javiana and Heidelberg are among the 20 most common among clinical cases in the US, I 4,[5],12:b:-, Thompson and Weltevreden less common but still among the 100 most common serotypes, whereas Okatie is rare with 0–6 annual clinical cases typically reported (https://www.cdc.gov/nationalsurveillance/pdfs/2016-Salmonella-report-508.pdf). The outbreak investigation was expanded to include these serotypes. No particular brand of the product could be implicated but the product was recalled from the market by several distributors and retailers, including on-line businesses. In total, 199 cases were identified from 41 states (https://www.cdc.gov/salmonella/kratom-02-18/index.html). Figures 3A,B shows cgMLST trees of representative isolates from patients and kratom of ser. I 4,[5],12:b:- and Okatie. Most of the I 4,[5],12:b:- isolates (Figure 3A) formed a tight subcluster with no more than one allele difference and this cluster lead to the identification of the source. However, another subcluster contained isolates differing by up to 25 alleles and an additional two isolates differing from the clustered isolates by up to 552 alleles. The ser. Okatie isolates were loosely clonal (Figure 3B). Of this serotype, 10 clinical isolates and 10 product isolates were sequenced differing from each other by up to 78 alleles; four subclusters were identified each containing isolates that differed by up to 2, 8, 9, and 13 alleles, respectively. Of note, each subcluster contained both clinical and product isolates.

The cgMLST results of the four other serotypes showed loose clustering in between what was seen with the two serotypes in the figures with more than 10 allele differences typically seen in monoclonal *Salmonella* outbreaks.

Kratom is grown and harvested in several countries in South East Asia and the sale and distribution systems are not transparent. Thus, it is possible that product for sale in the US originated from multiple producers in different countries and that the same product could contain kratom from more than one source. This likely explains why so many serotypes were involved. It may be speculated that the cluster caused by *Salmonella ser*. I 4,[5],12:b:- have recently contaminated kratom from one producer since it was tightly clonal, whereas the other serotypes may have been present in the production or distribution systems longer giving them time to diversify or have resulted in different contamination events at multiple producers. Because of the observed strain diversity with all serotypes it is unlikely that all clinical case-patients could have been identified without the availability of product isolates. However, the cluster associated with ser. Okatie could have been and actually was detected before the ser. I 4,[5],12:b:- cluster by serotype-based laboratory surveillance without considering WGS since it is so rare in the US. However, the association to kratom was not established before the serotype was detected in the product and the patients interviewed about that exposure. This serotype has scarcely been reported in the scientific literature but could have a focus in South East Asia.

## A Polyclonal Outbreak of Multidrug Resistant *Campylobacter* Linked to Contact With Puppies Sold in a Specific Pet Store Chain in the US

**Figure 4:**
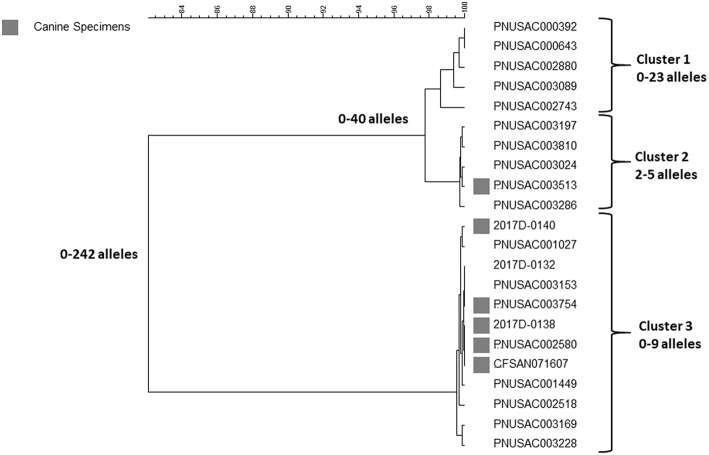
cgMLST UPGMA tree of a representative sample of sequences of clinical and animal isolates of *Campylobacter jejuni* from an outbreak associated with contact to puppies sold in a specific pet store chain. Puppy isolates are marked with gray squares. The range of allele differences are indicated at the branches of the tree and clusters to the right of the tree.

This outbreak was investigated and included illnesses reported over 2 years from 2016 to 2018 (https://www.cdc.gov/campylobacter/outbreaks/puppies-9-17/index.html). One-hundred and eighteen cases of illness caused by *Campylobacter jejuni* were identified in 18 states. The isolates were resistant to 7–9 antimicrobials including the drugs commonly used to treat patients with severe illness, e.g., azithromycin, ciprofloxacin and tetracycline. This particular multidrug resistant pattern was very rare in the US when compared to data from the National Antimicrobial Resistance Monitoring System (https://www.cdc.gov/narms/index.html). Infection was associated with contact to puppies sold in a specific pet store chain. Fifty-six clinical and puppy isolates were sequenced and analyzed by cgMLST using the PulseNet customized version of the Oxford scheme (24). A sample representing the full diversity observed in the outbreak is shown in Figure 4. At least three outbreak clusters were identified among the patient isolates. Two of the clusters (cluster 2 and 3) also contained puppy isolates.

In the cgMLST analysis for this paper, cluster 1 contained clinical isolates that differed by up to 23 alleles; the second cluster contained clinical and puppy isolates that differed by up to 8 alleles, and the last cluster also consisted of clinical and puppy isolates differing from each other by up to 28 alleles. All isolates were multidrug resistant as determined by WGS using ResFinder, which produced similar resistance profiles by phenotypic antimicrobial resistance testing examined on select isolates. For a small subset of isolates, long read sequencing was used to determine the genetic context of resistance determinants. These determinants were found to be located on the chromosome, or on a plasmid, or on both, or missing altogether. While some determinant's location, for example the *tetO* gene, tended to sort according to clonal group (plasmid for cluster 1 and 2, plasmid and chromosome for cluster 3), other genes' location, including several aminoglycoside resistance genes, did not sort by cluster. Moreover, at least one isolate had no plasmids but had all of the resistance determinants seen in this outbreak on its chromosome. Thus, there was no apparent correlation between plasmid content and resistance, but the resistance pattern itself was relatively stable among outbreak isolates.

## Multiple Outbreaks of Salmonellosis Linked to Small Pet Turtles, 2015–2016

**Figure 5:**
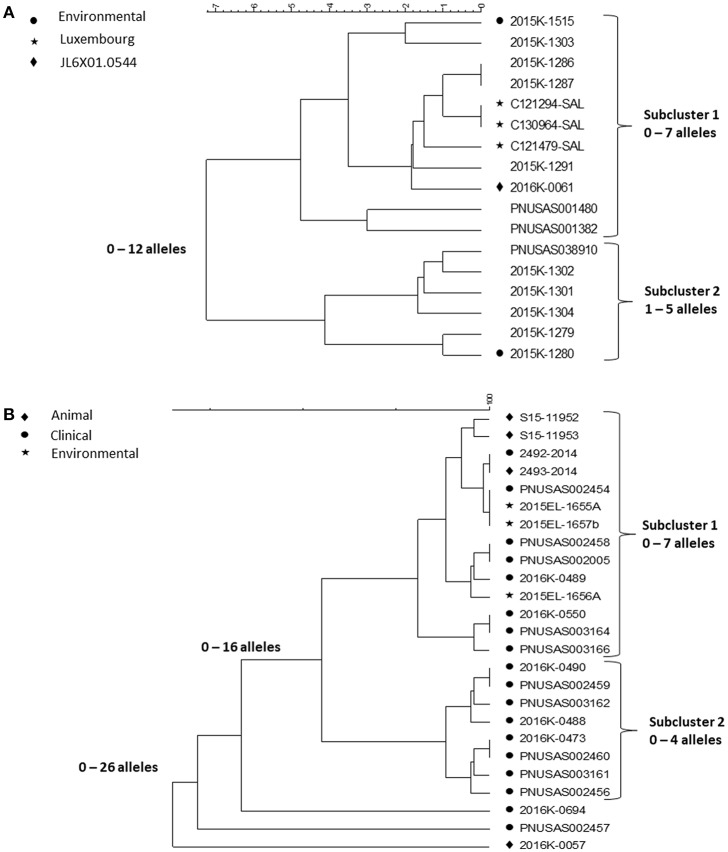
cgMLST UPGMA trees of representative samples of sequences from one of the *Salmonella* outbreaks associated with turtle exposure. **(A)** shows the relationships between isolates of ser. Poona; isolates from the environment, from Luxemburg or of a PFGE pattern different from that displayed by all other isolates in the figure are indicated next to the strain identifier. **(B)** shows the relationship between isolates of ser. Pomona; the source of the isolates is indicated next to the strain identifier. Isolates 2493-2014 and 2492-2014 are from Chile. The range of allele differences are indicated at the branches of the tree and subclusters to the right of the tree.

Contact to reptiles is a well-known risk factor for salmonellosis. Outbreaks associated with contact to small pet turtles are common [(25, 26), https://www.cdc.gov/salmonella/agbeni-08-17/index.html]. Their characteristics are similar and here we focus on four multi-state outbreaks caused by *Salmonella* in 2015**–**16 (26) and in particular, the WGS results in one of them, a polyclonal outbreak, caused by ser. Pomona and Poona. The investigation began as a follow-up on a consumer complaint about a child who had acquired a *Salmonella* infection from a small turtle acquired at a flea market of a serotype involved in turtle associated outbreaks years earlier (25). The PulseNet national database was checked for PFGE clusters the past year of serotypes previously linked to turtles. This way, four multistate outbreaks with 143 case patients from 25 states of three serotypes, Sandiego, Pomona and Poona, representing six PFGE patterns were identified. This outbreak investigation included testing of human, animal and environmental isolates. Nineteen *Salmonella* isolates were cultured from the pond water of four turtle production farms in Louisiana and from turtles and water tanks from eight cases. Since turtles from the US are exported all over the world, international inquiries and literature review were conducted resulting in the identification of one potential PFGE matching patient isolates in Chile and four in Luxembourg. The patients from Chile and two from Luxembourg had confirmed exposure to turtles. Of the 116 US patients with information available, 56 (48%) reported exposure to turtles. PFGE could not separate isolates from patients reporting contact to turtles from isolates from patients with no turtle contact. WGS was then used to test if isolates associated with different sources could be differentiated by this method. cgMLST results of isolates of ser. Poona and ser. Pomona from the biggest of the outbreaks [outbreak 2 in (26)] are shown in the Figures 5A,B.

The Poona isolates were loosely clustered in two tighter subclusters. Overall isolates differed by up to 12 alleles whereas isolates in the two tighter subclusters differed up to seven and five alleles, respectively. All isolates except one had the same PFGE pattern JL6X01.0104; one isolate, in the first subcluster, had a different PFGE pattern JL6X01.0554. Subcluster 1 contained isolates from Luxembourg, clinical isolates from the US and a turtle tank water isolate from a patient's home. The second subcluster also contained patient isolates from the US and one turtle tank water isolate. Whereas, the allele variation in each subcluster was <10 alleles typically observed in point source outbreaks, each of them could have been detected by WGS. Because they were less related between clusters, an association between them to the same source might not have been suspected without additional information, i.e., exposure information and/or a non-human isolate linking them to turtles. The isolates from Luxembourg were obtained in 2012 and 2013 indicating that the ser. Poona isolates from 2015 to 2016 had hardly evolved.

The Pomona isolates all displayed the same PFGE profile and formed two tight subclusters and three isolate that appeared unrelated to the two subclusters. The first subcluster contained clinical isolates from patients with turtle exposure and isolates from turtles, pond, and tank water; the subcluster also contained the patient and associated turtle isolate from Chile from 2014 (2493-2014 and 2492-2014 in the figure). The sequences in this subcluster differed from each other by up to seven alleles. Isolates in the second subcluster differed by up to four alleles. It only contained clinical isolates and all patients reported no turtle exposure. A common exposure between patients in this subcluster was never identified. The three non-clustered isolates were a turtle isolate (2016K-0057) from an earlier outbreak in 2012 and two current clinical isolates.

## Discussion

A well-functioning surveillance system that integrates elements from public and animal health and the food production is optimal to detect, investigate, and solve infections commonly transmitted through food (27). The examples provided in this paper illustrate that zoonotic outbreaks and outbreaks with a persistent environmental focus, which is typical for outbreaks in the One-Health context, are often not tightly monoclonal and may therefore be difficult to recognize through laboratory based surveillance by whole genome sequencing (WGS). This technology provides so much resolution that outbreaks that are caused by strains that have had time to evolve in the environment or in their natural hosts can be seen to have more variation than observed in typical point-source outbreaks. Using a One-Health approach in an integrated surveillance system, epidemiologic information, and isolates from animal and environmental sources, can greatly add to the ability to discriminate relatedness to clinical outbreak isolates. A number of different approaches may be used to detect, delineate, and investigate these outbreaks.

Considering additional information extracted from the sequencing information may help identify outbreaks, e.g., serotype information about an outbreak strain for a rare serotype such as *Salmonella* ser. Okatie that was associated with the outbreak linked to kratom described before. However, additional information may also cause confusion. For example, detailed information about resistance markers and plasmids can be confusing since these markers often not stable traits. However, despite such diversity multidrug resistance was helpful in recognizing and investigating two of the outbreaks described before: the *Salmonella* ser. Heidelberg associated with chicken from one production company, and the *Campylobacter* outbreak linked to pet store puppies. Similarly, PFGE may be used the same way. During the past 5 years, PFGE has remained the primary subtyping method in PulseNet with WGS used as a secondary confirmatory method except for *Listeria* where both methods have been used concomitantly for real-time surveillance. *Campylobacter* isolates are rarely subtyped in PulseNet unless an outbreak is suspected by other methods, e.g., like a cluster of multidrug resistant cases in the puppy outbreak. In the examples provided in this paper, PFGE mostly provided too much discrimination between isolates or the opposite, failed to differentiate isolates that were unrelated: multiple PFGE patterns were identified in the *Campylobacter* outbreak but only three clones were observed by WGS with so much variation in two of them that it would have been difficult to recognize them without additional resistance and exposure information. The outbreak was eventually confirmed by isolating the outbreak clones in pet store puppies and puppies owned by ill people. In the *Listeria* outbreak, enormous diversity was observed by PFGE, whereas WGS easily defined three outbreak clones/strains. In the turtle Poona outbreak subcluster described before, WGS helped differentiate PFGE clustered isolates from patients without contact to turtles from patients who had this exposure.

If a persistent or zoonotic focus for foodborne pathogens is suspected, the sequencing cluster definition may be relaxed. This may be done by initially looking for tight monoclonal clusters, e.g., differing by up to 10 alleles/SNPs, spanning a short time span since logically isolates from patients getting ill at the same time has a higher likelihood of originating from a point source, which could be a sub cluster of a larger zoonotic outbreak. Once the outbreak is recognized, and the initial patient interviews indicate that exposure to animals or an environmental source could be the vehicle, the case definition may be expanded in increments to include isolates that differ from the index cluster by for instance 25, 50, and 100 alleles or SNPs. Without associated epidemiological information, this approach may result in the inclusion of too many epidemiologically unrelated isolates during the outbreak investigation diluting any epidemiological signal that may be present. Therefore, foodborne, zoonotic, and environmental exposure information and isolates from food, zoonotic, and environmental sources should be used to determine different allele or SNP cutoffs choosing the values that provide the strongest epidemiological association. The utility of having access to sequencing information from potential sources is also extremely useful when working on an outbreak with a zoonotic or environmental focus. However, the ability to gather this information from animal isolates can be limited, as there often are few animal isolates available for comparison purposes during outbreaks, unless additional efforts are undertaken to collect them. This is at variance with clinical isolates, which are routinely collected by public health laboratories and sequenced to obtain additional information. Representative enteric bacterial isolates collected from animals are not routinely sequenced in the US. As shown in all the outbreaks described before, obtaining isolates from the potential sources was helpful to confirm the vehicle and also to facilitate recognition of the outbreak (the *Listeria* outbreak) or define its full scope (the ser. Heidelberg outbreak and the *Salmonella* outbreak linked to kratom). Thus, the importance of using a One-Health or farm to a table approach with efficient trace back when investigating outbreaks caused by pathogens commonly transmitted through food cannot be over emphasized.

International outbreaks caused by foodborne pathogens are common and WGS has the potential of bringing their recognition to the next level as more laboratories implement WGS in their routine surveillance. Until now most international outbreaks have been recognized by linking national outbreaks to each other when one country is investigating an outbreak with possible international spread and contacts other countries. Public health authorities in another country or countries may be contacted directly if there is a strong suspicion that the source of the outbreak is present in that country/those countries. Alternatively, the country may send out an inquiry through international rapid alert systems, e.g., the European RASSF system (https://ec.europa.eu/food/safety/rasff_en), or alert WHO through the IHR system (28). However, the information is more commonly shared broadly through listservs or data sharing boards, e.g., the European Center for Disease Prevention & Control (ECDC) EPIS system (29), the WHO INFOSAN (https://www.who.int/foodsafety/areas_work/infosan/en/) or the PulseNet International forum (30). The countries who receive this information are expected to report whether they are investigating a similar outbreak or see the frequency of the outbreak strain at a higher than usual rate in their surveillance of clinical and non-human surveillance isolates. If a country routinely uses a low discriminatory subtyping method for laboratory surveillance, e.g., species or serotype, this kind of comparison is insensitive and countries with one or a few outbreak related isolates are likely to overlook them. For instance, two of the outbreaks described here, the kratom and turtle-associated *Salmonella* outbreaks, were linked to globally distributed vehicles and yet, only two countries reported cases associated with turtle outbreak and no cases linked to kratom were detected outside the US. Another weakness of the international inquiry approach is that the comparison is not performed until an investigation is well under way in one country thereby delaying the investigation. Any country should ideally be able to access subtyping information on isolates from other countries in order to recognize international outbreaks fast. Except for the US and Canada who since 2005 have had access to each other's PulseNet databases, no other countries shared molecular surveillance data this way in real-time until the advent of WGS.

The potential of WGS to transform detection and investigation of international outbreaks was realized already in 2011 when scientists from what was later established as the Global Microbial Identifier (GMI) initiative met with the European commission in Brussels. The outcome of the meeting was published as a white paper (31). GMI envisions a global system of DNA genome databases for microbial and infectious disease identification and diagnostics fully embracing the One-Health concept. Sharing of surveillance sequence data with the global scientific community supports the mission of public health institutions and the One Health concept by facilitating early recognition and investigation of international outbreaks that a country is impacted by and therefore need to know about in order to act to protect its citizens. A global system for sharing of genomic data will benefit those tackling individual problems at the frontline, clinicians, veterinarians, environmental scientists, as well as policy-makers, regulators, and industry. By enabling access to this global resource, a professional response on health threats will be within reach of all countries with basic laboratory infrastructure (http://www.globalmicrobialidentifier.org/). PulseNet expanded on that vision in 2017 (32) suggesting a global system of databases containing data extracted from raw sequences of foodborne pathogens using standard analytical pipelines including the cgMLST pipelines used by PulseNet USA in this paper. The advantage of storing data extracted using standardized methods is 2-fold, (i) the data volume is greatly reduced enabling its exchange over slow internet connections, which is still the standard in many developing countries, and (ii) the data are standardized and can be used with minimal additional processing by any PulseNet participant ensuring fast comparison of data from databases in different regions of the world. Also, similar to PulseNet practices, realizing this global vision would likely be aided by additional laboratories submitting raw sequence files of all isolates obtained as part of routine surveillance in real-time to public repositories, e.g., the European Nucleotide Archive (ENA), the DNA Data Bank of Japan (DDBJ) or the GenomeTrakr databases in the Sequence Read Archive (SRA) at the National Center for Bioinformatics Information (NCBI). However, this is currently not possible for institutions in many countries for different reasons, e.g., protection of personal identifiable information (PII), intellectual property rights, or protection against scientific parasitism, i.e., publication of analyzed data generated by others without permission. The federal agencies in the US including CDC, FDA, and the US Department of Agriculture's (USDA's) Food Safety Inspection Service (FSIS) have uploaded all their raw sequences to the SRA in real time for the last 5 years without any noticeable adverse effects. An increasing number of agencies and institutions in other countries are now following suit, but there is still a long way to go before this is done by all countries.

## Conclusions

Outbreaks linked to animals and environmental sources can be challenging to recognize by laboratory surveillance by WGS because they are often polyclonal and more diverse than observed in typical point source outbreaks. The availability and use of supporting epidemiological information and microbiological information from non-clinical sources may be critical for their recognition and successful investigation. In the future, linking public health and food regulatory databases that include patient and food/feed/ingredient demographics, interview data, and microbiological data to national and international databases containing diverse types of other information, e.g., trade and distribution of different commodities, including live animals, raw agricultural products, processed foods, and international travel information, to name a few, could be used in a “big data” approach to detect and investigate outbreaks sometimes even before they become apparent by traditional syndromic or laboratory surveillance. However, critical first steps toward this vision include collection and sequencing of isolates from animal and environmental sources and all countries agree to make all their WGS surveillance data available to the others as they are generated before an outbreak is suspected.

## Author Contributions

PG-S conceived the paper and was the responsible writer. JC-A, JF, JH, LJ, ZK, and BT analyzed the sequences, interpreted the data, and contributed to the writing of the manuscript. JB, MN, and CS contributed with interpretation of the data and the writing of the manuscript.

### Conflict of Interest Statement

The authors declare that the research was conducted in the absence of any commercial or financial relationships that could be construed as a potential conflict of interest.
