# Corrigendum: Whole Genome Sequencing: Bridging One-Health Surveillance of Foodborne Diseases

**DOI:** 10.3389/fpubh.2019.00365

**Published:** 2019-12-06

**Authors:** Peter Gerner-Smidt, John Besser, Jeniffer Concepción-Acevedo, Jason P. Folster, Jasmine Huffman, Lavin A. Joseph, Zuzana Kucerova, Megin C. Nichols, Colin A. Schwensohn, Beth Tolar

**Affiliations:** ^1^The Enteric Diseases Laboratory Branch, Centers for Disease Control and Prevention, Atlanta, GA, United States; ^2^The Outbreak Response and Prevention Branch, Centers for Disease Control and Prevention, Atlanta, GA, United States

**Keywords:** whole genome sequencing (WGS), outbreak, one health, zoonotic, food, environment, animals, investigation

In the original article, there was a mistake in Figure 1 and Figure 2 as published. The graphics used are different than those originally submitted. The corrected Figure 1 and Figure 2 appear below.

**Figure 1:**
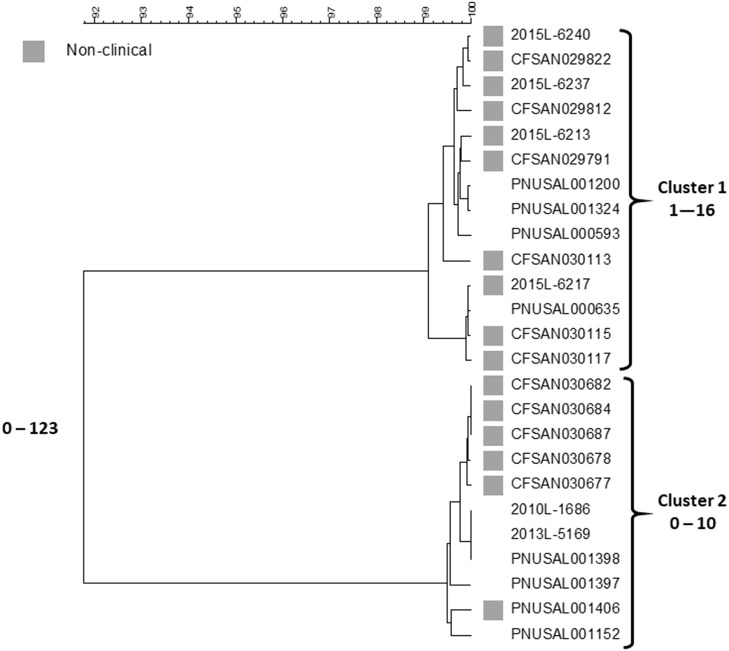
cgMLST UPGMA tree of Lineage I isolate sequences the Listeria outbreak linked to ice cream. All clinical isolates and a representative sample of non-clinical product and production environment isolates are included in the tree. The range of allele differences are indicated at the branches of the tree and for clusters to the right of the tree.

**Figure 2:**
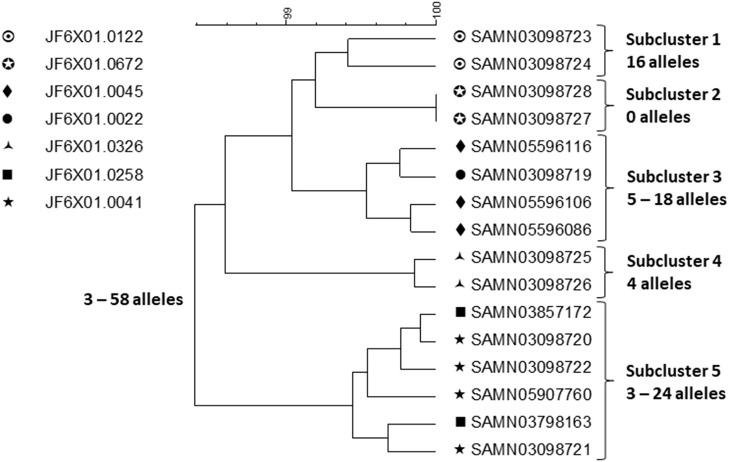
cgMLST UPGMA tree of a representative sample of sequences of Salmonella ser. Heidelberg isolates displaying the full WGS diversity and representing the seven PFGE patterns from the outbreak associated with chicken produced by Company A. The range of allele differences are indicated at the branches of the tree and subclusters to the right of the tree.

The authors apologize for this error and state that this does not change the scientific conclusions of the article in any way. The original article has been updated.

